# Second primary cancers among males with a first primary prostate cancer: a population-based study in Northern Portugal

**DOI:** 10.1007/s10238-025-01654-7

**Published:** 2025-04-21

**Authors:** José Taveira-Barbosa, Samantha Morais, Teresa Garcia, Maria José Bento, Nuno Lunet

**Affiliations:** 1 Departamento de Ciências da Saúde Pública e Forenses e Educação Médica, Faculdade de Medicina, Universidade do Porto, Al. Prof. Hernâni Monteiro, 4200-319 Porto, Portugal; 2 Serviço de Epidemiologia, Instituto Português de Oncologia do Porto FG, EPE, Porto, Portugal; 3 EPIUnit, Instituto de Saúde Pública da Universidade do Porto, Porto, Portugal; 4 Laboratório Para a Investigação Integrativa e Translacional em Saúde Populacional (ITR), Porto, Portugal; 5 Grupo de Investigação em Epidemiologia, Resultados, Economia e Gestão em Oncologia-Centro de Investigação (CI-IPOP) and Porto Comprehensive Cancer Center (Porto.CCC) and RISE@CI-IPOP (Rede de Investigação em Saúde), Instituto Português de Oncologia do Porto FG, EPE (IPO-Porto), Porto, Portugal; 6 Departamento Estudos de Populações, Instituto de Ciências Biomédicas Abel Salazar (ICBAS), Universidade do Porto, Porto, Portugal

**Keywords:** Second primary neoplasm, Prostatic neoplasm, Population register, Epidemiology

## Abstract

Increased survival and life expectancy among patients with prostate cancer results in an increased risk of developing a second primary cancer (SPC). We aimed to describe the occurrence of SPCs in a population-based cohort with a prostate first primary cancer (FPC), in Northern Portugal. A cohort of 13,222 patients with a prostate FPC from the North Region Cancer Registry of Portugal, diagnosed between 2000 and 2009, was followed until 31 December 2021, for synchronous (within six months of FPC diagnosis) and metachronous SPCs (all others). We describe absolute and relative frequencies of SPCs, incidence rates and standardized incidence ratios of SPCs (compared to the male general population), and cumulative incidence of metachronous SPCs. A total of 1953 (14.8%) patients with a prostate FPC developed an SPC, mostly of the colon, lung and bladder; synchronous SPCs occurred mainly in the bladder. Compared to the general male population, patients with a prostate FPC had a globally lower incidence of all cancers, and lung and oesophagus cancers, but a higher incidence of bladder and pancreas cancers. Overall, the incidence of synchronous SPCs was also significantly higher, likely reflecting the incidental diagnosis of SPCs. The 20-year cumulative incidence of metachronous SPCs was 15.4%. Patients with a prostate FPC had a lower overall incidence of SPCs than the general male population, despite a higher incidence in the first six-months after the SPC diagnosis. After that period, one out of seven may be expected to develop an SPC within two decades. Continued cancer surveillance among survivors is needed.

**Supplementary Information:**

The online version contains supplementary material available at 10.1007/s10238-025-01654-7.

## Introduction

Worldwide, prostate cancer (PCa) represents the second most common tumor diagnosed and ranks fifth in mortality among males in 2022 [1]. PCa is the most prevalent cancer with more than five million men alive five-years after diagnosis [1]. Furthermore, the worldwide incidence of PCa is expected to double by 2040 [2]. In Portugal, PCa ranks first in incidence and third in mortality among males, with an estimated 29-thousand males living with PCa five-years after diagnosis [1].

Survival from PCa has been increasing, with current estimates for 10-and 15-year relative survival at 98% and 91%, respectively [3], resulting from continuous improvements in diagnosis and treatments [4, 5]. Increased survival and life expectancy among patients with PCa results in increased risk for several adverse health events such as developing a second primary cancer (SPC), which might be associated with common genetic, environmental, lifestyle, etiological and treatment-related factors [6, 7].

The development of an SPC heavily impacts the survivorship journey of patients with PCa. Estimating the risk of developing an SPC and identifying the most common SPC sites is necessary to manage patient expectations regarding their prognosis and surveillance protocols.

In the present study, we aimed to quantify the incidence rates and risk of developing an SPC in a population-based cohort of patients from Northern Portugal with prostate first primary cancer (FPC) with a maximum of 22 years of follow-up, and to compare the incidence of SPCs among patients with prostate FPC with the expected in a sex-, age- and calendar year-matched population.

## Methods

### Study setting (study population and data source)

Cancer data were obtained from the population-based Registo Oncológico Regional do Norte/North Region Cancer Registry (RORENO). The registry was established in 1988 and covers the Northern region of Portugal, accounting for approximately 3.5 million inhabitants, which is nearly one-third of the Portuguese population [8]. RORENO registration follows the International Agency for Research and Cancer (IARC) rules including four quality dimensions: comparability, validity, timeliness, and completeness. RORENO maintains quality through regular screening with pre-defined algorithms for validity and consistency, having fulfilled IARC indices of data quality between 1998 and 2002 [9]. RORENO calculates cancer incidence using estimates of the resident population in the area covered by the registry each year, according to Statistics Portugal [8]. The results are expressed as an annual rate per 100,000 person-years [8].

### Tumour classification and definition of second primary cancers

The International Classification of Diseases for Oncology, Third Edition [ICD-0–3][10] was used to classify tumour topography and morphology, and then recoded to the International Statistical Classification of Diseases and Related Health Problems 10th Revision [ICD-10][11]. SPCs were defined according to the guidelines proposed by the International Association of Cancer Registries (IACR) and IARC [12]. SPCs are new primary cancers in a person with a history of malignancy that originally developed in an organ or tissue not being an extension, recurrence, or metastasis [12]. Different morphologies (even with the same topography) or dissimilar topographies were considered as multiple primary cancers, regardless of the time between diagnoses, unless they correspond to systemic cancers [12].

### Study design

**Fig. 1:**
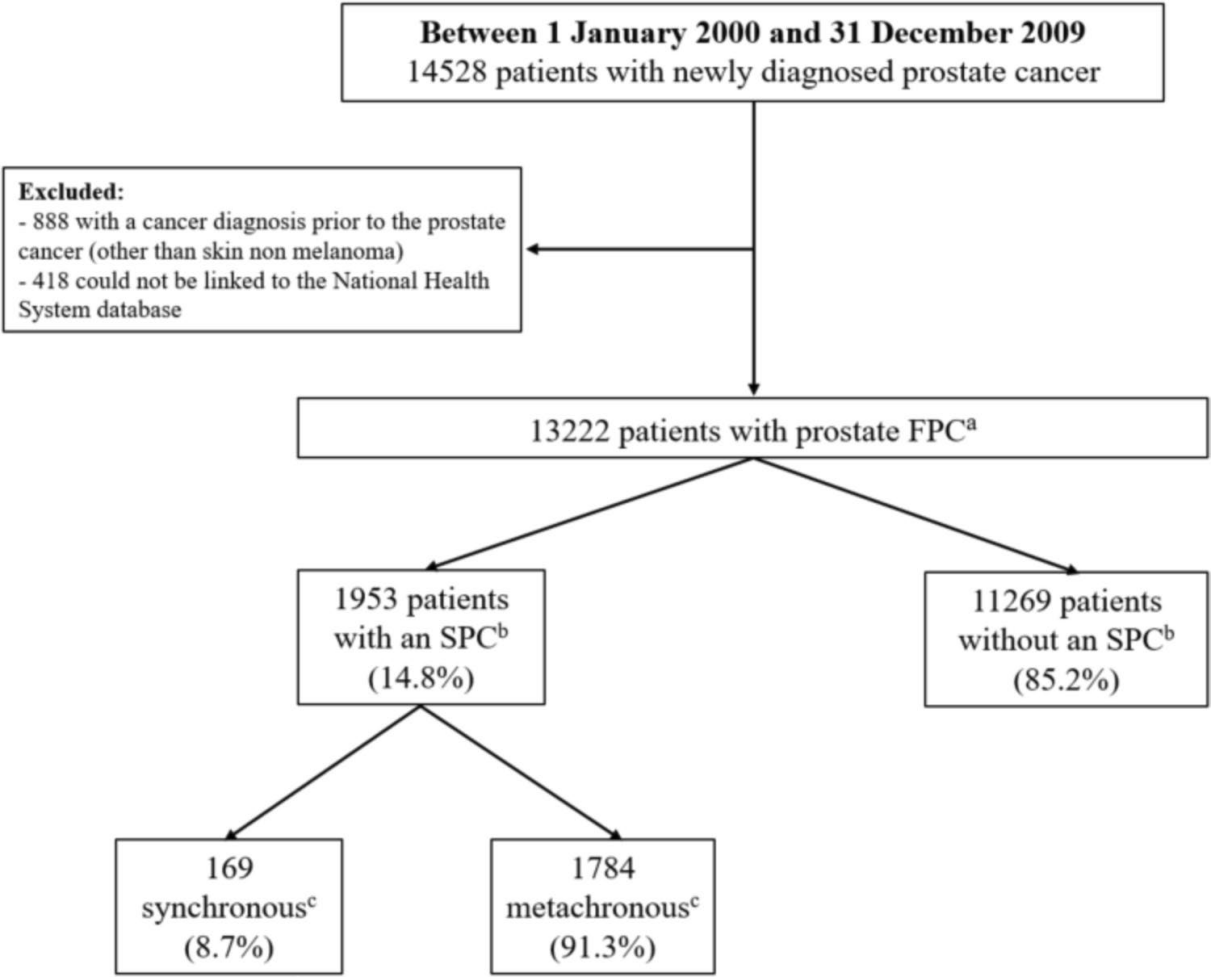
Flow chart of patient inclusion in the study (follow-up for identification of second primary cancers and death up until 31 December 2021). ^a^FPC–first primary cancer. ^b^SPC–second primary cancer. ^c^Synchronous—diagnosed within the first six months of the prostate FPC, remaining classified as metachronous

All primary invasive tumours of the prostate (C61) diagnosed in adult male residents in the North of Portugal between 1 January 2000 and 31 December 2009 were identified (n = 14,528) (Fig. 1). Patients who had a cancer diagnosis, except skin non-melanoma, previous to the PCa (n = 888) and those who could not be linked to the National Health System database for assessment of vital status (n = 418) were excluded (Fig. 1).

The remaining patients (n = 13,222) were followed to 31 December 2021, allowing for 12 to 22 years of potential follow-up for a diagnosis of a subsequent primary cancer or death, whichever occurred first. The occurrence of any SPC was ascertained by linkage with the registered cases by RORENO. Only the SPC was considered; third and subsequent primaries were disregarded in this analysis.

Due to the thorough evaluation of patients with cancer during the initial medical work-up, SPCs were classified as synchronous when diagnosed within six months of the prostate FPC and metachronous otherwise, according to the proposed rules by the Associazione Italiana Registri Tumori/ Italian Network of Cancer Registries (AIRTUM) for defining synchronous cancers [13].

### Statistical analyses

Patients’ characteristics are presented as counts and proportions for categorical variables, and as medians (percentile 25-percentile 75 [P25-P75]) for continuous variables. The Chi-square test and the Mann–Whitney test were used to compare categorical and continuous variables across groups, respectively. Statistical significance was considered for p < 0.05, and all reported p-values are two-sided. Analyses were carried out separately for synchronous and metachronous SPCs.

The incidence rate of SPCs was computed by dividing the number of incident SPCs by the person-years at risk (PYAR) in the whole male population, for different follow-up periods (“0 to < 1 month”–from FPC diagnosis to less than one month; “ ≥ 1 to < 2 months”, and so on during the first year of follow-up; “ ≥ 12 to < 18 months”–from 12 to less than 18 months; “ ≥ 18 to < 24 months, and so on to the sixth year of follow-up; and then for each year of the follow-up). PYAR were calculated as the time from prostate FPC diagnosis to the SPC diagnosis, death or end of follow-up (31 December 2021), whichever occurred first.

To compare the incidence of SPCs in patients with prostate FPC with the expected incidence in an age- and calendar year-matched population, standardized incidence rations (SIRs) were calculated by dividing the observed number of SPCs by the expected number of cases, in the same time period, if the cancer incidence rates in the general male population had been observed among survivors of prostate FPC. The latter were estimated by multiplying the cancer incidence in the general male population by the PYAR in the corresponding stratum defined according to five-year age group (from 15-19 to 70–74, and ≥ 75 years for 2000–2006; and from 15-19 to 80–84, and ≥ 85 years from 2007 to 2021) and calendar year (2000–2021). The cancer incidence (overall and for the most frequent SPC sites: stomach (C16), colon (C18), rectum (C19-C20), pancreas (C25), trachea, bronchus and lung (C33-C34), kidney (C64), bladder (C67) and Non-Hodgkin lymphoma (C82-C86, C96)) among the general male population was obtained from RORENO’s annual publications on cancer incidence in the general population [8]. SIRs were estimated for all cancers (excluding prostate (C61) cases from the expected number of SPCs, as developing a prostate SPC following a prostate FPC is a very rare event) and separately for synchronous and metachronous SPCs. 95% confidence intervals (CIs) for the SIRs were estimated assuming that the number of cancer cases followed a Poisson distribution.

Cumulative incidence of metachronous SPCs and corresponding 95% CIs, stratified by age, were calculated, considering death as a competing event according to the method of Kalbfleisch and Prentice [14]. The observed cumulative mortality was estimated using 1—Kaplan–Meier [15].

Additionally, sensitivity analyses were performed defining synchronous SPCs as diagnosed two months and one year after the prostate FPC and excluding urological (bladder and kidney) cancers in the estimation of all, synchronous and metachronous SIRs.

## Results

Among 13,222 patients with a prostate FPC diagnosed between 2000 and 2009, 1953 (14.8%) developed an SPC during the follow-up until 31 December 2021, of which 169 (8.7%) were considered as synchronous (diagnosed within the first six months of the prostate FPC), and the remaining metachronous. Patients who developed an SPC were generally younger at prostate FPC diagnosis compared to those without an SPC (respectively 80.1% vs. 72.0% of patients younger than 75 years old). The median age at FPC diagnosis was 69 years. Those who had a synchronous SPC were older at prostate FPC diagnosis. The median time follow-up was over 16 years for all groups of patients, and the median time between FPC and metachronous SPC diagnosis was over six years (Table 1).

**Table 1:** Characteristics of patients with prostate cancer without a second primary cancer and with synchronous and metachronous second primary cancers

	Total	Patients without an SPC	Patients with an SPC						
	N = 13,222	N = 11,269	N = 1953						
			Total	*P value*	Synchronous	*P value*	Metachronous	*p-value*	*p-value*
				All SPC vs. no SPC	n = 169	Synchronous SPC vs. no SPC	n = 1784	Metachronous SPC vs. no SPC^a^	Synchronous SPC vs. Metachronous SPC
Age at diagnosis of PCa, years [median(P25-P75)]	69(63–75)	69(63–75)	69(63–73)	** < 0.001**	72(66–78)	**0.001**	68(63–73)	** < 0.001**	** < 0.001**
< 60	1846(14.0%)	1587(14.1%)	259(13.3%)		24(14.2%)		235(13.2%)		
60–64	2009(15.2%)	1683(14.9%)	326(16.7%)		15(8.9%)		311(17.4%)		
65–69	2860(21.6%)	2376(21.1%)	484(24.8%)		27(16.0%)		457(25.6%)		
70–74	2963(22.4%)	2469(21.9%)	494(25.3%)		38(22.5%)		456(25.6%)		
75–79	2191(16.6%)	1921(17.1%)	270(13.8%)		32(18.9%)		238(13.3%)		
≥ 80	1353(10.2%)	1233(10.9%)	120(6.1%)	** < 0.001**	33(19.5%)	**0.001**	87(4.9%)	** < 0.001**	** < 0.001**
Follow-up, years [median(P25-P75)]^b^	16.1(14.2–18.5)	16.1(14.1–18.5)	6.0(2.7–9.8)	** < 0.001**	0.2(0.1–0.4)	** < 0.001**	6.5(3.6–10.2)	** < 0.001**	** < 0.001**
**Dead**[N(%)]^c^	7777(58.8%)	6349(56.3%)	1428(73.1%)	** < 0.001**	131(77.5%)	** < 0.001**	1297(72.7%)	** < 0.001**	0.177

Significant associations are bolded

PCa – prostate cancer, P25—percentile 25, P75—percentile 75, SPC—second primary cancer

^a^Patients with no SPC in this comparison are those who survived more than six months after diagnosis of the first primary cancer

^b^Follow-up in years until: SPC, death or end of follow-up in 31 December 2021, whichever occurred first. Median follow-up was estimated using the reverse Kaplan–Meier

^c^Follow-up until 31 December 2021

### Incidence rate of second primary cancers and standardized incidence ratios

**Fig. 2:**
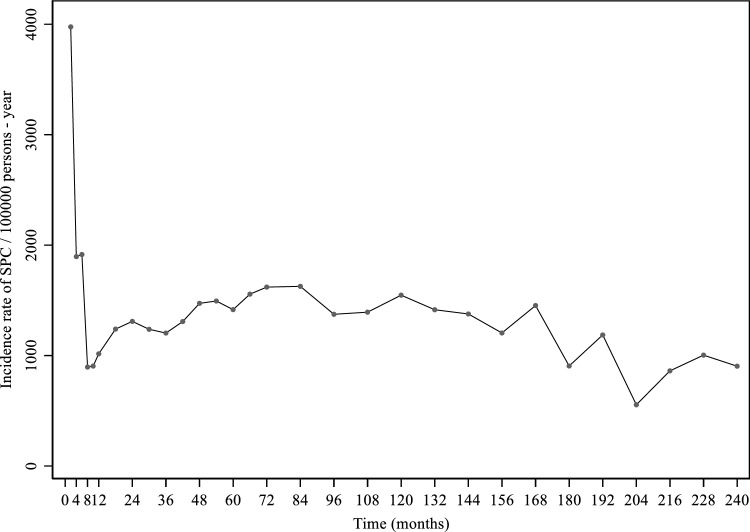
Incidence rates of second primary cancers (SPC) by time (in months) since diagnosis of the first primary prostate cancer. Incidence rates were estimated and are represented in the endpoint of the following intervals: each month in the first year of follow-up, every six months from the second year on to sixth year of follow-up, and then for each year of follow-up

The incidence rate of SPCs was around 4000 SPCs/100,000 person-years in the first two months of follow-up, decreasing to around 2000 SPCs/100,000 person-years for the following six months. From the first year until the 14th year of follow-up, incidence rates remained relatively stable with 1400 SPCs/100,000 person-years. From the 15th year (180 months) until the end of follow-up there was a decrease in the incidence rate to around 1000 SPCs/100,000 person-years (Fig. 2).

**Fig. 3:**
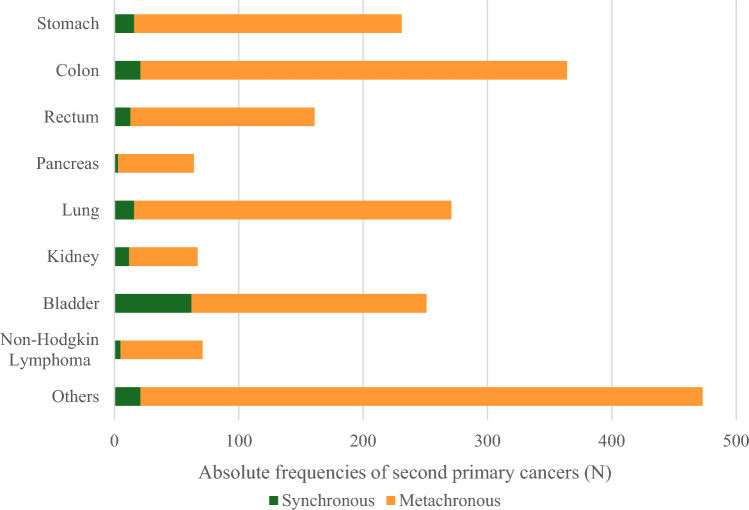
Absolute frequencies of second primary cancers by synchronous (six months since first primary cancer diagnosis) and metachronous (all others) and by most frequent cancer sites, among males with a first primary prostate cancer. Cancer sites include: Stomach (C16), Colon (C18), Rectum (C19-C20), Pancreas (C25), Trachea, bronchus and lung (C33-C34), Kidney (C64), Bladder (C67) and Non-Hodgkin lymphoma (C82-C86, C96). Others include all categories with less than 40 cases: Lip, oral cavity and pharynx (C00-C14), Oesophagus (C15), Small intestine (C17), Anus (C21), Liver and intrahepatic bile ducts (C22), Biliary tract (C24), Nasal cavity (C30), Larynx (C32), Bone (C41), Skin melanoma (C43), Mesothelial and soft tissue (C45-C49), Breast (C50), Penis (C60), Testis (C62), Renal pelvis (C65), Ureter (C66), Eye (C69), Brain and central nervous system (C70-C72), Thyroid (C73), Endocrine gland (C75), Without specification of site (C80), Hodgkin Lymphoma (C81), Immunoproliferative diseases (C88), Multiple myeloma (C90), Lymphoid leukaemia (C91), Myeloid leukaemia (C92-C94) and Other neoplasms of uncertain or unknown behaviour of lymphoid, haematopoietic and related tissue (D47) [11, 12]

The most frequent SPCs were observed in the colon, lung, bladder, stomach and rectum. Most synchronous SPCs occurred in the bladder, followed by the colon, lung, stomach and rectum (Fig. 3). Bladder had the highest proportion of synchronous vs. metachronous cancers (24.7%), followed by kidney (17.9%) and rectum (8.1%), while the lowest proportions were observed for pancreas (4.7%), colon (5.8%) and stomach (6.9%).

**Fig. 4:**
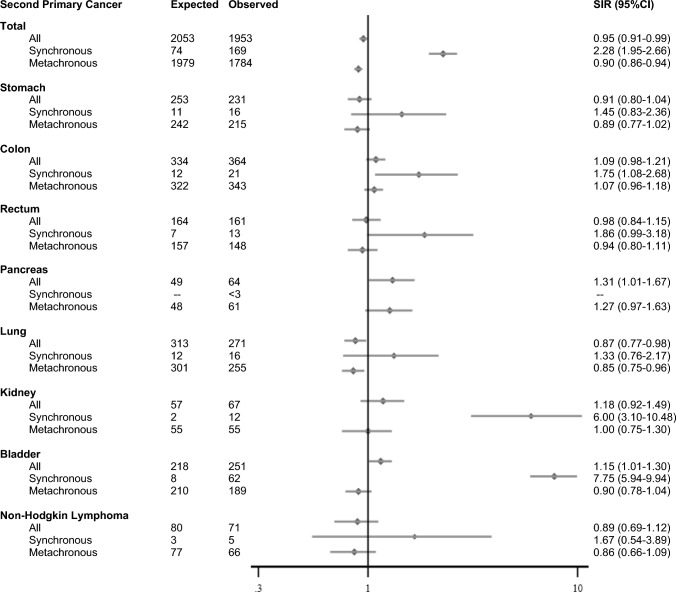
Standardized incidence ratios and 95% confidence interval, for the most frequent second primary cancers in patients with a first primary prostate cancer. 95% CI–95% confidence interval, SIR–standardized incidence ratios. Second primary cancers include: Stomach (C16), Colon (C18), Rectum (C19-C20), Pancreas (C25), Lung (C33-C34), Kidney (C64), Bladder (C67), Non-Hodgkin lymphoma (C82-C86, C96) [11, 12]

Overall, patients with a prostate FPC had a lower incidence of cancer compared to the general male population (SIR = 0.95; 95%CI: 0.91–0.99). SIRs were statistically significantly higher for synchronous SPCs (SIR = 2.28; 95%CI: 1.95–2.66) and conversely statistically significantly lower for metachronous SPCs (SIR = 0.90; 95%CI: 0.86–0.94) (Fig. 4).

Increased SIRs were observed for all SPCs for the bladder (SIR = 1.15; 95%CI: 1.01–1.30) and the pancreas (SIR = 1.31; 95%CI: 1.01–1.67). As well as for the colon (SIR = 1.09; 95%CI: 0.98–1.21) and the kidney (SIR = 1.18; 95%CI: 0.92–1.49) although these were not statistically significant. Decreased SIRs were observed for the lung (SIR = 0.87; 95%CI: 0.77–0.98). For synchronous SPCs, increased statistically significant SIRs were observed for the bladder (SIR = 7.75; 95%CI: 5.94–9.94), the kidney (SIR = 6.00; 95%CI: 3.10–10.48), and the colon (SIR = 1.75; 95%CI: 1.08–2.68) (Fig. 4).

### Cumulative incidence of metachronous second primary cancers and mortality

**Fig. 5:**
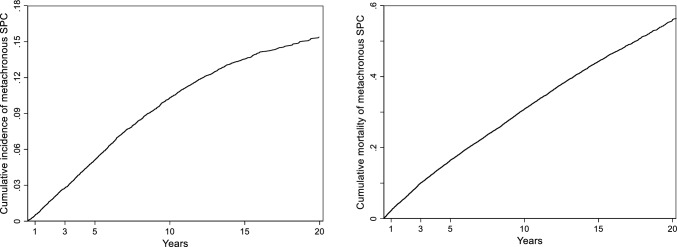
Cumulative incidence of metachronous second primary cancers (left) and corresponding cumulative mortality (right), among males with prostate first primary cancer. SPC, second primary cancer. *Note that a different scale is used for the two outcomes

The 10- and 20-year cumulative incidence for metachronous SPCs following a prostate FPC, was 10.3% (95%CI: 9.8–10.8) and 15.4% (95%CI: 14.7–16.1), respectively (Fig. 5). The corresponding 10- and 20-year cumulative mortality was 30.8% (95%CI: 30.0–31.6) and 56.0% (95%CI: 54.8–57.3). The cumulative incidence of metachronous SPCs at 5-, 10-, 15- and 20-years was generally higher between 60 to 74 years, while cumulative mortalities were higher for older age groups (Supplementary Table 1).

### Sensitivity analyses

When defining synchronous SPCs as occurring within two, six and 12 months of a prostate FPC, SIRs for synchronous SPCs were 3.48 (95%CI: 2.79–4.29), 2.28 (95%CI: 1.95–2.66) and 1.90 (95%CI: 1.66–2.16), respectively (Supplementary Table 2). Considering the complementary definition for metachronous SPCs, SIRs at two, six and 12 months for metachronous SPCs were 0.92 (95%CI: 0.88–0.96), 0.90 (95%CI: 0.86–0.94) and 0.89 (95%CI: 0.85–0.93), respectively (Supplementary Table 2). The 10- and 20-year cumulative incidence of metachronous SPCs was 10.7%, 10.3% and 10.1% when defining metachronous SPC as two, six and 12 months after the prostate FPC, respectively.

When excluding urological (bladder and kidney) cancers from the analysis, the SIRs for all, synchronous and metachronous SPCs were, respectively, 0.92 (95%CI: 0.88–0.97), 1.48 (95%CI: 1.20–1.81) and 0.90 (95%CI: 0.85–0.94).

## Discussion

In the present study, patients with a prostate FPC had a lower incidence of cancer compared with the general male population. The majority of SPCs occurred in the colon, followed by lung, bladder and stomach, while most synchronous SPCs occurred in the bladder. The cumulative incidence of metachronous SPCs was 10.7% and 15.7% at 10- and 20-years, and corresponding cumulative mortalities were 31.7% and 56.3%.

To the best of our knowledge, this is the first Portuguese population-based study with a focus on SPCs among patients with a prostate FPC. Several population-based cancer registry studies have been published on the development of SPC among patients with a prostate FPC with heterogeneous results [16–20]. The proportion of patients with a prostate FPC that develop an SPC varies widely among different studies, settings, methodologies and follow-up times. In our cohort, 14.8% of patients developed an SPC during a median follow-up of 16 years. In the United States (US), 10.0% of men diagnosed with a prostate FPC from 1992 to 2010, developed an SPC during the same period [6]. In Sweden, 11.3% of patients with a prostate FPC diagnosed between 2001 and 2010, had a subsequent SPCs diagnosis after a median follow-up of 4 years [17]. While in Switzerland, 8.4% of patients diagnosed with a prostate FPC from 1980 to 2010 developed an SPC during the same period [18]. In Korea, 4.7% men diagnosed with a prostate FPC between 1993 and 2011 developed an SPC during the same period [19]. The higher proportion of SPCs identified in our cohort compared to previous studies, is likely associated with a longer median follow-up time, a result of the study design which allowed us to have a minimum follow-up of 12 years for patients diagnosed with a prostate FPC.

In our study, the 10-year cumulative incidence of metachronous SPCs was 10.7%. Few studies have estimated cumulative incidences for SPCs among patients with PCa. The Danish Cancer Registry reports an overall 10-year cumulative incidence of SPCs in patients with prostate FPC of 12.8%, which was highest for SPCs of the lung (1.9%), colon (1.8%) and bladder (1.6%) [20].

The most frequent sites for SPCs observed were the colon, lung, bladder and stomach, which is in line with results from previous studies. In the US and Switzerland, the lung, bladder and colon were the most frequent SPC sites [6, 18]. A Swedish population-based registry study identified colorectal, skin, bladder, lung, melanoma, and non-Hodgkin lymphoma as the most common SPCs [17]. In a Korean cancer-registry study the most common SPCs were diagnosed in the stomach, colon, lung and bladder [19]. Patterns of SPC among patients with a prostate FPC are highly concordant with those expected for the general male population in Portugal [8]. SPCs might occur due to common etiological risk factors, such as obesity, tobacco consumption, occupational exposure, high blood pressure, as well as hormonal exposures and genetic predisposition or common pathways of carcinogenesis [4, 21–23]. Moreover, the occurrence of SPCs may reflect the late effects of cancer treatments, namely radiotherapy, a common curative treatment for PCa. Large cohort studies exploring SPC risk after PCa have suggested that radiotherapy is associated with increased SPC risk, and in PCa specifically SPCs of the bladder [4, 24, 25]. While direct radiation carcinogenesis has long been accepted, there is evidence that irradiation of the prostate might contribute to carcinogenesis outside the irradiated area through radiation scatter [26, 27]. As a result, the inclusion of pelvic lymph nodes in standard radiation therapy for PCa treatment is a significant controversy topic and its application is at the discretion of the treating physician [28]. In our study, information on individual patients’ treatment regime was not available, thus we were not able to differentiate patients who underwent radiation therapy treatment for PCa from those who did not. However, the high number of synchronous SPCs of the bladder observed are largely explained by screening frequency, anatomical proximity and overlap of symptoms and diagnostic tests for both cancers.

Previous studies report contrasting findings regarding SIRs of cancer among patients with PCa compared to the general male population. In our study, we observed statistically significant lower SIRs of 0.95 (95%CI: 0.91–0.99). A lower SIR was estimated when PCa cases were included in the expected number of cases (SIR = 0.71; 95%CI: 0.68–0.75). While the reasons behind the reduction in risk are unclear, assuming that a large proportion of the cases of PCa are diagnosed after screening [29] we may hypothesize a “healthy screenee bias”, with the individuals undergoing cancer screening being more likely to be less exposed to risk factors, such as smoking. A systematic review [30] and a pooled study from five Swedish cohorts [31], report an inverse association between smoking consumption and incidence of PCa, with smoking associated with lower risk of PCa, likely due to low uptake of prostate-specific antigen testing by smokers. The lower proportion of smokers among patients with PCa compared to the general male population, could explain the decreased SIRs for lung (SIR = 0.87; 95%CI: 0.77–0.98) and oesaphagus (SIR = 0.66; 95%CI: 0.43–0.96) cancers observed in our study, as smoking is a known risk factor for both pathologies [32]. Further, smoking cessation post-PCa FPC diagnosis is also likely to play a factor in reducing the risk of developing an SPC of the lung. Several studies [32–34] have reported an increased risk of lung SPCs in patients who continued to smoke, compared to those who stopped smoking. Thus, we should not neglect the potential effects of smoking cessation in the decreased on observed SPCs in our cohort.

In the US, a cohort of more than 440 thousand men followed between 1992 and 2010, reported an SIR of 0.60 (95%CI: 0.60–0.61). With increased SIRs for bladder (SIR = 1.05; 95%CI: 1.02–1.07) and kidney (SIR = 1.12; 95%CI: 1.07–1.17) cancers [6]. In Switzerland, a cohort of more than 20 thousand men followed between 1980 and 2010, reported an SIR of 1.11 (95%CI: 1.06–1.17), with increased SIRs for site-specific cancers of the colon (SIR = 1.73; 95%CI: 1.34–2.19) and bladder (SIR = 1.97; 95%CI: 1.52–2.52) [18]. In Korea, a cohort of more than 55 thousand men diagnosed between 1973 and 2011 with primary PCa had an overall lower incidence of SPC (SIR = 0.75; 95%CI: 0.72–0.78), there were, however, significant increases in the incidence of bladder (SIR = 1.26; 95%CI: 1.09–1.45) and thyroid cancers (SIR = 2.06; 95%CI: 1.63–2.56) [19]. The observed differences could be due to differences in the time periods covered, lengths of follow-up, study designs, and patient populations. Studies with a short duration of follow-up are unlikely to capture late toxicity effects from the FPC treatment [35]. The classification of synchronous cancers has not been based on homogeneous criteria, and some studies present results including metachronous SPCs only [6, 18]. Different methodologies are also used for defining the follow-up of diagnosis between an FPC and the subsequent SPC. Often studies do not have different periods for diagnosis, resulting in the inclusion of patients with very short follow-up times, who are, by definition, less likely to develop an SPC. Further, different methodologies to calculate SIRs result in different results, as reported by the French cancer registries. An analysis on SIRs including and excluding SPCs in the same cancer site as the FPC, reported a SIR of 1.11 (95%CI: 1.07–1.14) for SPCs in patients with a prostate FPC when the occurrence of a second cancer of the prostate was excluded; when this was not performed, the SIR dropped to 0.72 (95%CI: 0.69–0.74) [36]. In our study, to avoid this bias, we excluded expected prostate SPCs from the SIR calculation. While most PCas are adenocarcinoma, a small number of other morphological types can arise (e.g. neuroendocrine tumours or mesenchymal tumours), thus it is not impossible to observe an SPC of the prostate in patients with a prostate FPC [36]; this situation, however, did not arise in our study.

Further, observed differences could also be a result of different definitions of multiple primary cancers. The definitions and understanding of multiple primary cancers have changed over time and are likely to differ among studies, especially for different study periods. Currently the two most common definitions used are provided by the Surveillance Epidemiology and End Results (SEER) project and the IARC, with differences in the definition of subsequent cancers. One of the main differences is that several groups of topography codes of ICD-10 are considered one site in the definition of multiple primaries (IARC 2004) [13, 37, 38]. The definition of synchronous and metachronous multiple primaries and its inclusion on the calculation and reporting of the SIRs also plays a significant role in the observed differences. Considering the above, we presented all, synchronous and metachronous SIRs separately and used an interval of six months. SEER recommends the use of a two-month period to distinguish between synchronous and metachronous multiple primaries, while AIRTUM suggests a six-month period to distinguish between synchronous and metachronous multiple primaries [13, 38]. To facilitate comparisons with other studies, we conducted sensitivity analyses considering different criteria to define SPCs.

### Strengths and limitations

This study is based on data from the population-based registry of Northern Portugal, RORENO, which is representative of patients with PCa in the region and covers one-third of the country’s population. We followed patients for a minimum of 12-years and more than two decades, thus we were able to capture a significant period of risk for developing subsequent malignancies.

As information on clinical data was not available, namely FPC and SPC stage at diagnosis, treatment regimens (radiation therapy, surgery, etc.), family history, genetic susceptibility and individual lifestyle risk factors, we were unable to quantify their contribution to the development of SPCs.

## Conclusion

In our study based on data from Northern Portugal, we observed that male patients with PCa have a lower incidence of SPCs compared to the general male population. Nevertheless, our findings suggest that patients with PCa remain at risk of developing subsequent cancers. The development of subsequent malignancies among PCa survivors poses a burden on the individual level and healthcare resources. Continued cancer surveillance among PCa survivors is needed.

## Supplementary Information

Below is the link to the electronic supplementary material.

Supplementary file1 (DOCX 16 kb)

## Data Availability

The data that support the findings of this study are available from RORENO, but restrictions apply to the availability of these data, which were used under license for the current study and so are not publicly available. Data are however available from the authors upon reasonable request and with permission of RORENO.
