# Supplementary material for: Second primary cancers among males with a first primary prostate cancer: a population-based study in Northern Portugal
Source: Clin Exp Med. 2025 Apr 21;25(1):122. doi: 10.1007/s10238-025-01654-7 (PMC12011927; doi:10.1007/s10238-025-01654-7)
Supplement: Supplementary file 1 — Supplementary file1 (DOCX 16 kb) [file 10238_2025_1654_MOESM1_ESM.docx]

**Supplementary Table 1**. Cumulative incidence (top) and cumulative mortality (bottom) of metachronous second primary cancers following a prostate first primary cancer, estimated at 5-, 10-, 15- and 20-years by age group at first primary cancer diagnosis (<60; 60-64; 65-69: 70-74; 75-79; ≥80 years)

|  |  |  |  |  |
| --- | --- | --- | --- | --- |
| **Cumulative Incidence** | **5 years** | **10 years** | **15 years** | **20 years** |
| **<60** | 3.5 (2.7-4.4) | 8.0 (6.7-9.2) | 12.2 (10.6-13.7) | 15.0 (13.1-17.0) |
| **60-64** | 4.5 (3.6-5.4) | 10.3 (8.9-11.6) | 15.0 (13.4-16.6) | 17.9 (15.8-19.9) |
| **65-69** | 5.4 (4.5-6.2) | 11.3 (10.1-12.4) | 15.6 (14.2-17.0) | 18.7 (16.9-20.6) |
| **70-74** | 6.5 (5.6-7.4) | 12.6 (11.4-13.8) | 15.6 (14.3-17.0) | 17.1 (15.6-18.6) |
| **75-79** | 5.3 (4.3-6.3) | 9.8 (8.6-11.1) | 11.4 (10.0-12.8) | 11.5 (10.1-12.9) |
| **≥80** | 4.5 (3.3-5.6) | 6.9 (5.5-8.4) | 7.2 (5.8-8.7) | 7.2 (5.8-8.7) |
|  |  |  |  |  |
| **Cumulative Mortality** | **5 years** | **10 years** | **15 years** | **20 years** |
| **<60** | 6.5 (5.4-7.7) | 11.5 (10.1-13.0) | 17.5 (15.7-19.3) | 23.9 (21.1-26.6) |
| **60-64** | 8.1 (6.9-9.3) | 15.3 (13.7-16.8) | 24.2 (22.3-26.1) | 33.3 (30.3-36.2) |
| **65-69** | 9.8 (8.7-10.9) | 20.4 (18.9-21.9) | 34.8 (33.0-36.6) | 51.3 (48.4-54.3) |
| **70-74** | 15.1 (13.8-16.4) | 31.3 (29.6-33.0) | 49.9 (48.0-51.8) | 66.7 (64.1-69.3) |
| **75-79** | 25.6 (23.8-27.5) | 50.4 (48.2-52.5) | 68.5 (66.4-70.6) | 79.5 (77.2-81.8) |
| **≥80** | 47.6 (44.8-50.5) | 74.6 (72.2-77.1) | 83.9 (81.8-86.0) | 86.1 (84.1-88.2) |

**Supplementary Table 2.** Standardized incidence ratios for all, synchronous and metachronous second primary cancers following a first primary cancer, estimated for different cutoffs for synchronous cancers (2, 6 and 12 months)

|  |  |  |  |
| --- | --- | --- | --- |
|  | **Standardized Incidence Ratio** | | |
|  | All | Synchronous | Metachronous |
| **2 months** | 0.95 (0.91-0.99) | 3.48 (2.79-4.29) | 0.92 (0.88-0.96) |
| **6 months** | 0.95 (0.91-0.99) | 2.28 (1.95-2.66) | 0.90 (0.86-0.94) |
| **12 months** | 0.95 (0.91-0.99) | 1.90 (1.66-2.16) | 0.89 (0.85-0.93) |
|  |  |  |  |

**Supplementary Table 3.** Cumulative incidence and cumulative mortality of metachronous second primary cancers following a first primary cancer, estimated for different cutoffs for synchronous cancers (2, 6 and 12 months) and different time estimates (5-, 10-, 15- and 20-years)

|  |  |  |  |  |  |  |  |  |  |  |
| --- | --- | --- | --- | --- | --- | --- | --- | --- | --- | --- |
|  |  | **Cumulative Incidence** | | | |  | **Cumulative Mortality** | | | |
|  |  | 5 years | 10 years | 15 years | 20 years |  | 5 years | 10 years | 15 years | 20 years |
| **2 months** |  | 5.6 (5.2-6.0) | 10.7 (10.2-11.2) | 13.9 (13.3-14.5) | 15.7 (15.0-16.4) |  | 17.6 (16.9-18.2) | 31.7 (30.9-32.5) | 44.8 (43.9-45.7) | 56.3 (55.1-57.5) |
| **6 months** |  | 5.1 (4.7-5.5) | 10.3 (9.8-10.8) | 13.5 (12.9-14.1) | 15.4 (14.7-16.1) |  | 16.4 (15.8-17.1) | 30.8 (30.0-31.6) | 44.2 (43.3-45.1) | 56.0 (54.8-57.3) |
| **12 months** |  | 4.8 (4.4-5.1) | 10.1 (9.6-10.6) | 13.4 (12.8-14.0) | 15.3 (14.6-16.0) |  | 14.6 (14.0-15.2) | 29.4 (28.6-30.2) | 43.2 (42.3-44.1) | 55.3 (54.1-56.6) |
|  |  |  |  |  |  |  |  |  |  |  |
